# Design and validation of depth camera-based static posture assessment system

**DOI:** 10.1016/j.isci.2023.107974

**Published:** 2023-09-21

**Authors:** Qingjun Xing, Ruiwei Hong, Yuanyuan Shen, Yanfei Shen

**Affiliations:** 1 School of Sport Science, Beijing Sport University, Beijing 100084, China; 2 School of Sport Engineering, Beijing Sport University, Beijing 100084, China

**Keywords:** Sports medicine, Computer science, Engineering

## Abstract

Postural abnormalities have become a prevalent issue affecting individuals of all ages, resulting in a diminished quality of life. Easy-use and reliable posture assessment tools can aid in screening for and correcting posture deviation at an early stage. In this study, we present a depth camera-based static posture assessment system to screen for common postural anomalies such as uneven shoulders, pelvic tilt, bowlegs and knock-knees, forward head, scoliosis, and shoulder blade inclination. The system consists of an Azure Kinect camera, a laptop, and evaluation software. Our system accurately measures skeleton and posture indexes and shows favorable agreement with a golden standard optical infrared motion capture system. The findings indicate that the system is a low-cost posture assessment tool with high precision and accuracy, suitable for initial screening of postural abnormalities in individuals of all ages.

## Highlights

• We have developed a depth camera-based static posture assessment system
• Our system can detect common postural anomalies
• The precision and accuracy of our system are high compared to the gold standard

## Graphical abstract

**Figure:**
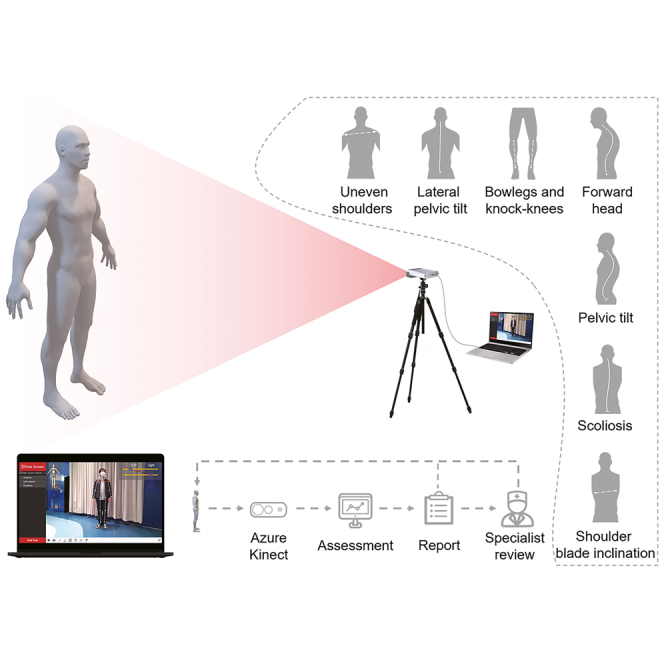

## Introduction

Body posture, as shown by the human body in the completion of basic activities such as sitting, standing, and walking without deliberate control,^1^ is a crucial physical health indicator. Good posture not only improves body image but also contributes to overall wellbeing. Conversely, poor posture is a manifestation of sub-health and is widespread,^2^ caused by long-term effects of unhealthy modern lifestyles, such as video games, fast food, lack of regular physical activity, prolonged use of handheld mobile devices and sedentary jobs.^2^^,^^3^ Unfortunately, there has been a trend of younger age groups exhibiting abnormal posture due to the nature of their musculoskeletal system. In Iran,^4^ China,^5^ Poland,^6^ New Zealand,^7^ and other countries, the abnormal posture detection rate of children aged from 3 to 18 years of age reached 45–78.6%. Common postural deviations found in children and adolescents include knee hyperextension, bowlegs and knock-knees, anterior pelvic tilt and pelvic obliquity, scoliosis, winged shoulder blades, uneven shoulders, and thoracic hyperkyphosis.^8^ Studies have shown that poor posture in childhood can lead to negative consequences in adulthood.^5^^,^^9^

**Figure 1:**
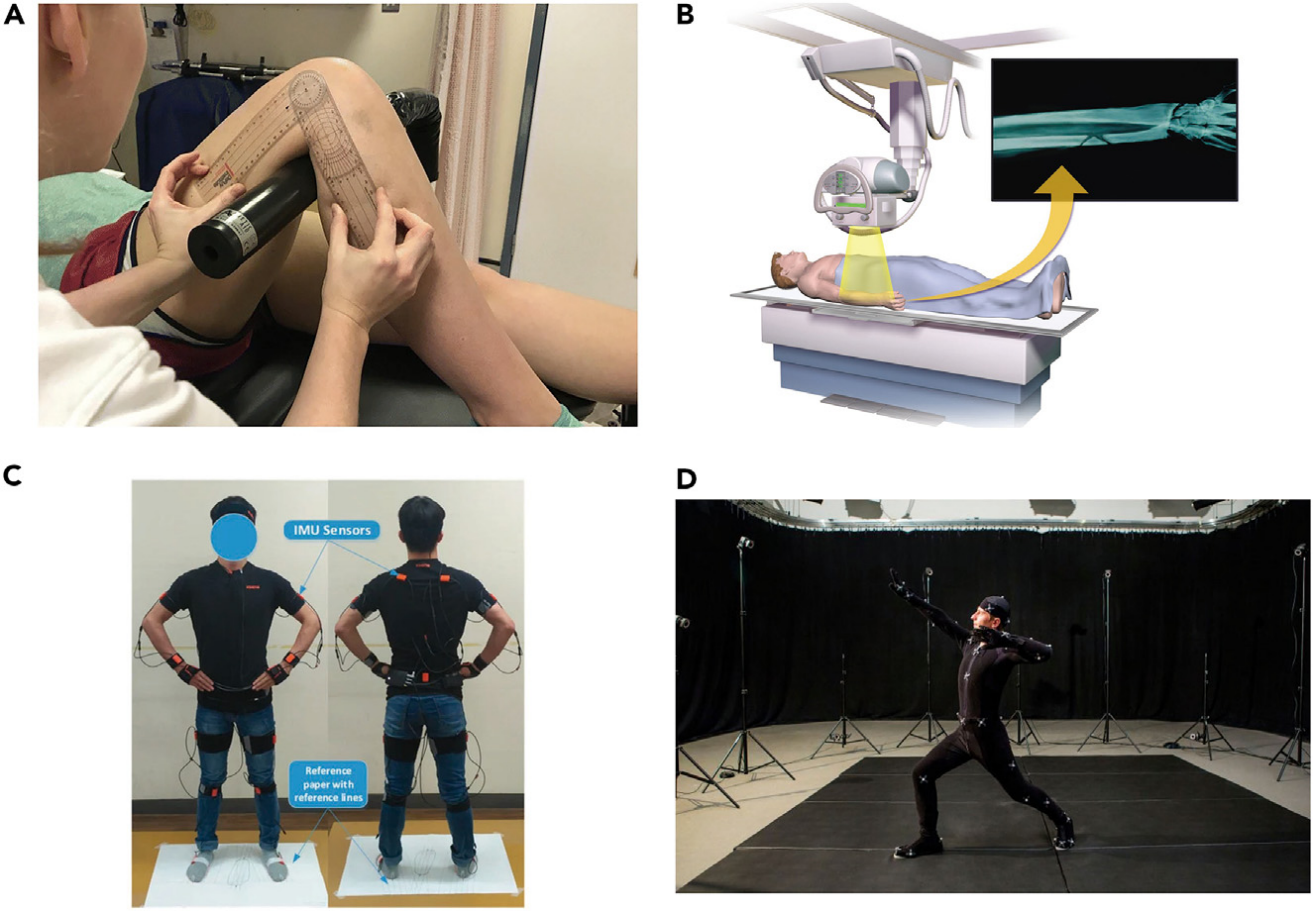
Several representative posture assessment methods from previous studies (A) Goniometry.^12^ (B) Radiography.^36^ (C) Inertial sensor based Xsens MVN.^24^ (D) Optical motion capture system.^37^

In recent decades, numerous posture assessment methods and tools have been proposed to address the above issue. These methods range from traditional visual observation techniques to equipment-assisted and automated non-invasive approaches. While traditional visual inspection and palpation techniques, such as flexiruler^10^^,^^11^ and goniometry (Figure 1A),^12^ are still employed in clinical practice,^13^ their reliability and reproducibility are low and depend on the skill of the assessor.^14^^,^^15^ As a result, these methods are not commonly used in the scientific and educational settings.^16^

Radiography is considered the gold standard for assessing spinal curvature (Figure 1B);^17^ its captured radiographic image provides essential information for accurate diagnoses of spinal abnormalities. This method enables analysis of individual vertebrae and the overall contour of the spine. However, radiography has a major disadvantage in its high cost and the risk of exposure to harmful radiation.^18^^,^^19^ Although an ultrasound system is radiation-free, it is not a suitable tool for a school environment due its weight, cost, dependence on a skilled operator, and time-consuming assessment of the entire spine.^20^^,^^21^

Over the last two decades, inertial sensors-based methods (Figure 1C)^22^^,^^23^^,^^24^ and photogrammetric-based^25^^,^^26^ approaches for postural assessment have been widely reported in the literature due to advances in sensor technology, including accelerometers, gyroscopes, flexible angular sensors, sensing fabrics and image sensors. In general, inertial sensors-based tools have been used in two application scenes. One is the kinematics analysis of dynamic actions; for example, using an algorithm based on double integration of inertial measurement units (IMUs) for high-accuracy gait analysis that can evaluate short and normal strides and is suitable for gait monitoring in daily life situations.^27^ The other is flexible electronics, which have been used in many studies to detect and correct postural deviations.^28^^,^^29^ However, these tools are complex to wear and operator-dependent, especially with low utilization of flexible electronics.

Photogrammetric-based methods, which are classified into two-dimensional (2D) and three-dimensional (3D) solutions, have been widely utilized in various applications. In 2D solutions, reflective markers are taped on anatomical reference points and limb segments, and photographs of the subjects are obtained from three different planes: anterior, posterior, and lateral. The results of the postural evaluation are then derived from accompanying software, such as Fizyoprint,^30^ PostureScreen,^31^ and SagittalMeter Pro.^32^ Although 2D solutions are easy to use, they do not provide a complete description of the 3D posture information. Therefore, 3D solutions have been used in many studies for postural analysis, as they are expected to offer more kinematic information about posture. Marker-based optical infrared motion capture systems, such as Vicon, OptiTrack, and Qualisys, are considered the gold standard for precise postural evaluation and motion tracking (Figure 1D).^33^^,^^34^^,^^35^ Their 3D positions can be reconstructed using triangulation with submillimeter accuracy and precision, based on the positions of passive/active markers in multiple camera images. However, these motion capture systems are expensive, complex, limited to laboratory environments, and require a time-consuming calibration process.

With the continuous advanced of depth sensor’s accuracy, RGB-Depth sensors have emerged as an alternative to optical infrared motion capture systems in some scenarios, such as gait analysis,^38^ postural control,^39^ rehabilitation exercise monitoring and guidance.^40^ These sensors are identified as an affordable, portable and marker-less motion capture devices that can be used in various indoor environments. Microsoft released Azure Kinect in 2019, which is an RGB-depth camera that utilizes the time of flight (ToF) principle and offers significantly higher accuracy than other consumer-grade depth cameras. Moreover, the Azure Kinect utilizes a new human body tracking software development kit (SDK), based on Convolutional Neural Networks (CNN), which has been used in many studies.

**Figure 2:**
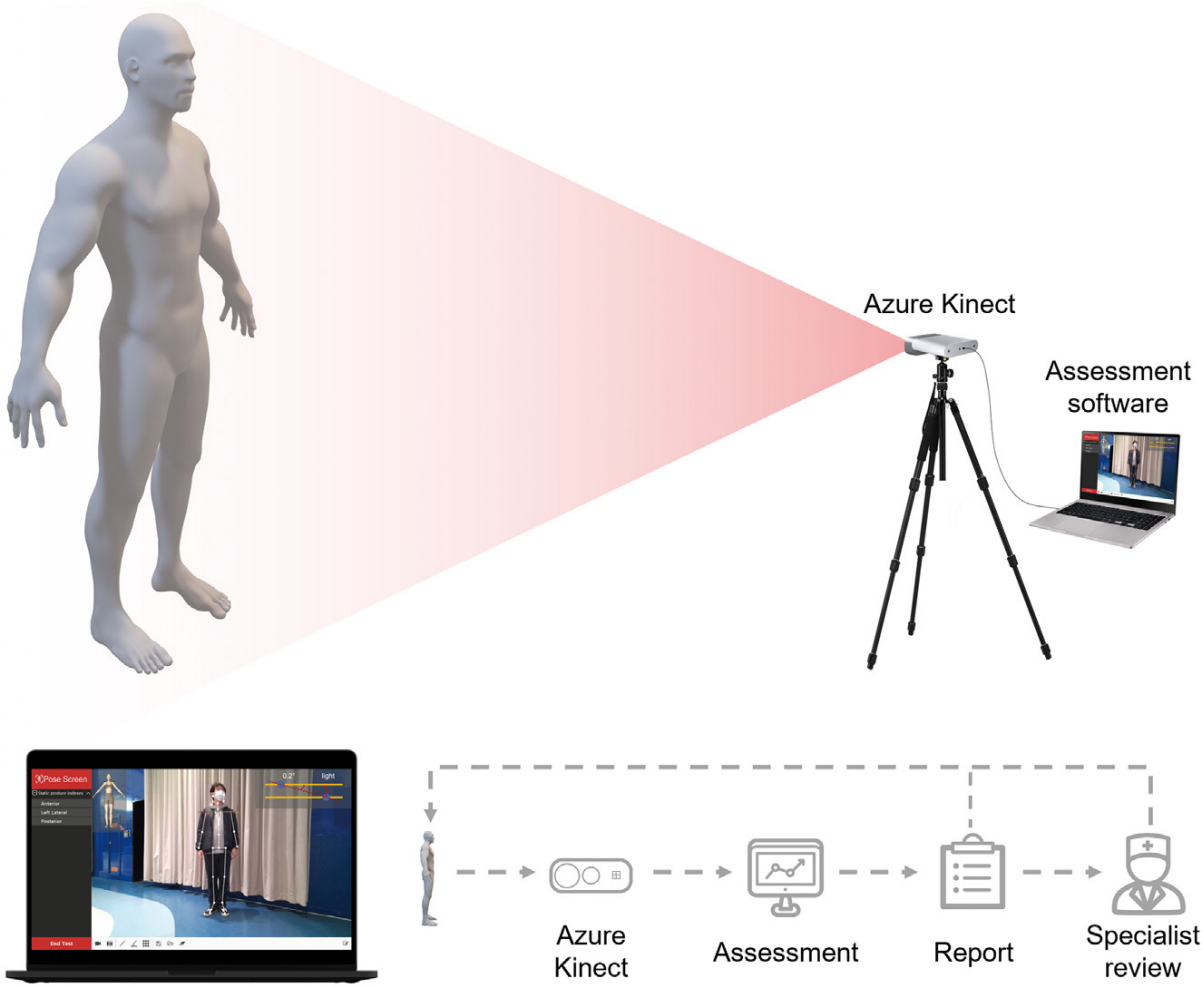
Overview of the postural evaluation system (Upper panel) A simulated scenario of the subject’s anterior static posture assessment using the system. (Lower panel) Evaluation test interface, and block diagram showing the potential application of the system in assisting specialist with initial screening for the general population.

**Figure 3:**
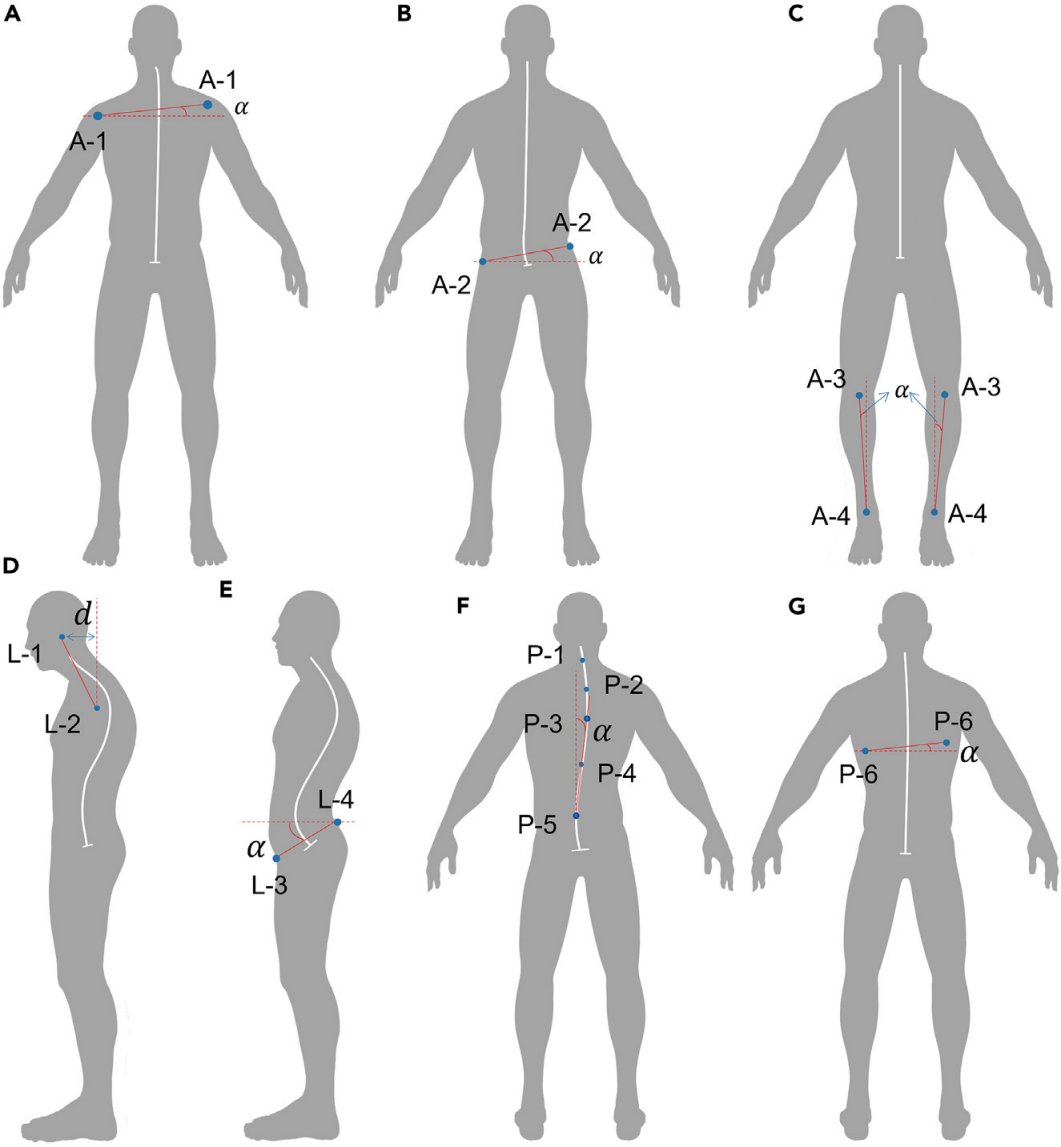
Postural test indexes in our system (A) Uneven shoulders. (B) Lateral pelvic tilt. (C) Bowlegs and knock-knees. (D) Forward head. (E) Pelvic tilt. (F) Scoliosis. (G) Shoulder blade inclination. A: Anterior; L: Lateral; P: Posterior. Marker positions notes: A-1: Left/Right Acromion; A-2: Left/Right Iliac Crest; A-3: Left/Right Patella; A-4: Left/Right Malleolus; L-1: Earlobe; L-2: Acromion; L-3: Anterior superior iliac spine (ASIS); L-4: Posterior superior iliac spine (PSIS); P-1: 7^th^ cervical vertebra (C7); P-2: Upper T-Spine; P-3: Mid T-Spine; P-4: Lower T-Spine; P-5: Lumbar 1 (L1); P-6: Left/Right scapula inferior angle (SIA).

Against this background, this study developed and validated an Azure Kinect-based posture evaluation system to aid operators in the preliminary screening postural deviation (Figure 2). The proposed system can evaluate common abnormal postures, such as uneven shoulders, pelvic tilt, bowlegs and knock-knees, scoliosis, shoulder blade inclination and forward head (Figure 3), with just three images taken from anterior, left lateral and posterior. The proposed system also offers several other features: (1) the use of only a few auxiliary markers pasted on the subject’s posterior anatomical reference points, (2) an intuitive graphical user interface (GUI), (3) real-time display of results, (4) the flexibility for operators to re-select anatomical reference points on the software interface using a mouse, and (5) report generation and export capability. Furthermore, the precision and accuracy of our system were validated against a gold standard, the OptiTrack motion capture system.

## Results

### System design

Our static postural evaluation system consists mainly of an Azure Kinect depth camera, a laptop, and an evaluation software developed using the Qt cross-platform application and GUI framework. The camera is placed horizontally on a tripod and connected to the laptop via a full-featured C to C cable, which supplies both power and data transfer. To evaluate posture, the operator only needs to obtain photographs from three planes: anterior, left lateral, and posterior. The system can automatically identify the subject’s anatomical joints; however, for some landmarks on the lateral and posterior that cannot be automatically identified, the operator could manually correct them using a mouse on the software screen. The system also supports saving subject’s images and skeleton data for later modification, as well as the generating and exporting evaluation reports. Figure 2 depicts a diagram of the system.

The posture measurement indexes that our system can evaluate are shown in Figure 3, and the scoring criteria of each posture indexes are shown in Table 1.

**Table 1:** Scoring criteria of each index in posture test

Measurement Index		Light	Medium	Heavy
Anterior	uneven shoulders	0°≤α < 2°	2°≤α < 4°	α ≥ 4°
	lateral pelvic tilt	0°≤α < 2°	2°≤α < 4°	α ≥ 4°
	bowlegs and knock-knees	−1°≤α < 3°	3°≤α < 5° (O Leg) −3°≤α < −1° (X Leg)	α ≥ 5° (O Leg) α ≤ −3° (X Leg)
Lateral	forward head	d < 2.5 cm	2.5 cm≤d < 5 cm	d ≥ 5 cm
	pelvic tilt	0°≤α < 15°	15°≤α < 25°	α ≥ 25°
Posterior	scoliosis	0°≤α < 2°	2°≤α < 4°	α ≥ 4°
	shoulder blade inclination	0°≤α < 5°	5°≤α < 10°	α ≥ 10°

### Software reliability as a static postural evaluation

#### Skeleton tracking precision of Azure Kinect

**Figure 4:**
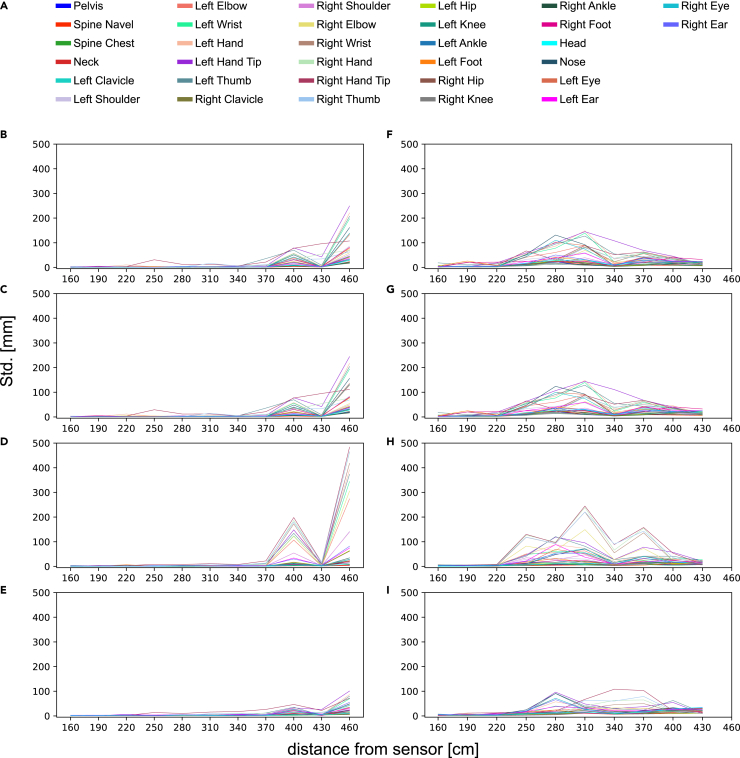
Azure Kinect’s skeleton tracking precision at different distances (A) The 32 joint names of the skeleton identified by Azure Kinect. Legend of this figure, as well as the legend of Figures 6 and 7. (B–E) Represents the Std. of Euclidean distance, depth value, X axis value and Y axis value of 32 joints without OptiTrack system interference at different distances, respectively. (F–I) Represents the Std. of Euclidean distance, depth value, X axis value and Y axis value of 32 joints with OptiTrack system interference at different distances, respectively.

**Figure 5:**
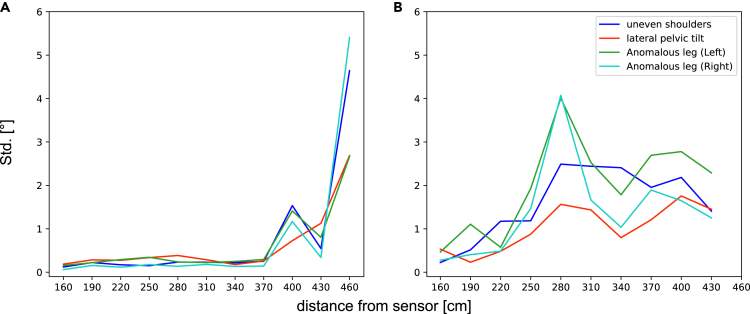
Anterior postural measurement indexes precision at different distances (A) The Std. of the posture indexes of the human anterior at different distances without OptiTrack system interference. (B) The Std. of the posture indexes of the human anterior at different distances with OptiTrack system interference.

The skeleton tracking precision of Azure Kinect was evaluated at different distances, with or without interference from the optical motion capture system, as shown in Figure 4 in the form of standard deviation (Std.). The results show that the highest precision was achieved when the camera was positioned between 160 and 220 cm away from the human body. The Std. of some joints decreased as the distance increased, but with varying degrees of oscillation. When there was no interference, Azure Kinect performs was able to track joints accurately up to 340 cm from the human body. However, in the presence of interference, the Std. of some joints significantly degraded at 250 cm. Furthermore, the camera was unable to identify the human body at 460 cm. In addition, through the analysis of Figure 4 (B-E), it can be seen that the instability of some joints is mainly manifested in depth values and X axis values. The Std. of different joints varied at the same distance, and this characteristic become more pronounced with the increase of distance. Meanwhile, similar results were observed for the human body anterior posture indexes, as shown in Figure 5. The precision of the anterior indexes is satisfactory up to 370 cm in the absence of interference from the optical motion capture system.

Table 2 presents the numerical data of Figure 4, including the average, maximum, and minimum Std. of the Euclidean distance between 32 joints captured by the camera at different distances with and without interference. See Data S1 for more detailed data.

**Table 2:** Euclidean distance’s Std. of 32 joints measured by Azure Kinect at different distances with and without interference (mm)

		160	190	220	250	280	310	340	370	400	430	460
Non-interfering	Max Std.	3.9	5.0	10.3	31.1	12.5	16.2	6.7	35.9	79.2	96.3	249.1
	Min Std.	0.4	0.5	0.7	0.5	0.7	0.6	0.6	0.6	3.6	1.3	17.3
	Avg Std.	1.2	1.6	2.0	2.3	2.1	2.4	2.1	3.9	27.7	9.5	80.0
Interfering	Max Std.	18.6	25.9	21.5	66.9	131.3	148.4	107.9	68.8	45.3	32.2	–
	Min Std.	1.5	1.4	1.6	6.4	13.2	8.6	3.6	8.0	8.2	6.4	–
	Avg Std.	3.7	5.1	5.4	24.9	44.2	55.4	18.0	31.8	22.7	15.8	–

**Figure 6:**
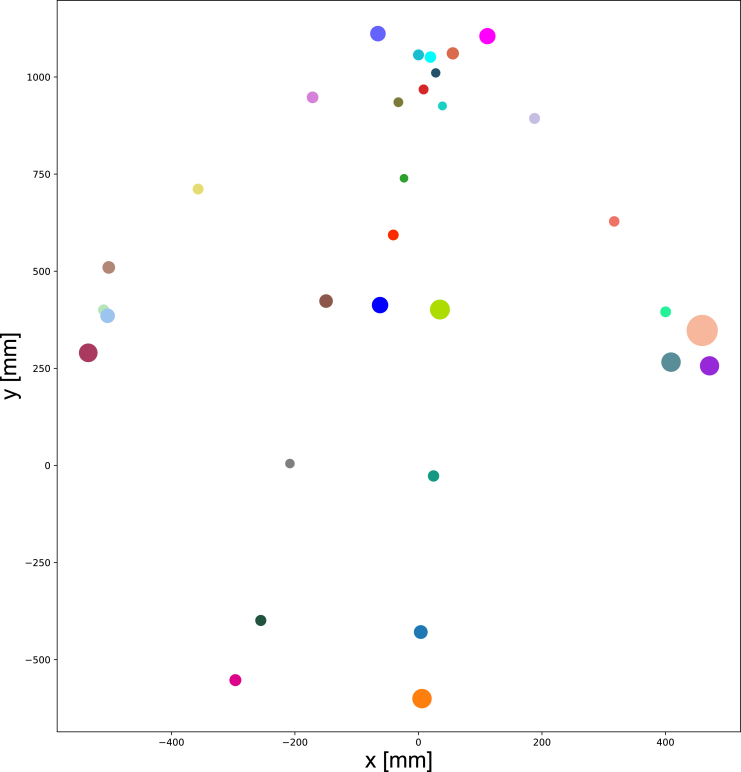
32 joints’ precision identified by Azure Kinect at same position (220 cm) without OptiTrack system interference

Next, we analyzed the precision of 32 joints at 220 cm from the human body, as shown in Figure 6. The size of each circle represents the Std. of a body joint 3D position multiplied by 100 for better visual clarity. Data from other positions with or without interference yielded similar results. It is found that joints located at the end of limbs, such as the hand tip, foot, and ear, generally had larger Std. values. This finding suggests that these joints are more challenging to track accurately.

#### Skeleton tracking accuracy of Azure Kinect

**Figure 7:**
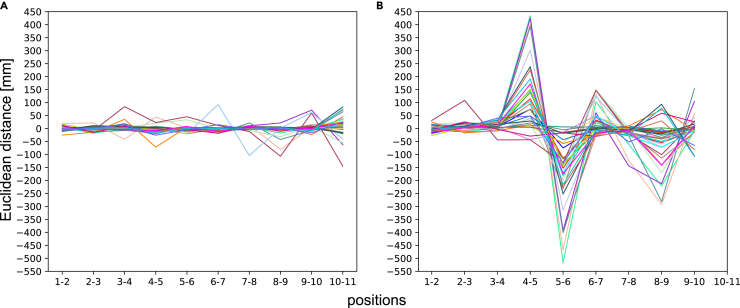
Skeleton tracking relative accuracy of Azure Kinect at different distances Compare the adjacent positions in pairs (A) without OptiTrack system interference. (B) with OptiTrack system interference.

In this section, we did not perform a rigid transformation on the two coordinate systems. Therefore, we are discussing relative accuracy rather than absolute accuracy. Relative accuracy refers to the distance difference between two adjacent positions. In addition, due to the possibility of errors in manually moving the Azure Kinect, we subtract the distance difference between the two positions in the OptiTrack geodetic coordinate system, as shown in Figure 7. Similar to the skeleton tracking precision test, the skeleton tracking accuracy of Azure Kinect is better when there is no interference from the OptiTrack system. Although the Azure Kinect provides good accuracy for skeleton tracking stability between 160 cm and 370 cm away from the human body in the absence of interference, Figure 7A shows that 160 cm–220 cm is still the optimal choice interval. Table 3 shows the numerical data of Figure 7. See Document S1 for more detailed data.

**Table 3:** Skeleton tracking relative accuracy of 32 joints measured by Azure Kinect at different distances with and without interference (mm)

		1–2	2–3	3–4	4–5	5–6	6–7	7–8	8–9	9–10	10–11
Non-interfering	Max dis	319	322	379	349	344	394	321	319	374	388
	Min dis	274	282	253	234	278	283	196	191	280	160
	Avg dis	299	299	301	298	299	301	298	290	308	314
Interfering	Max dis	331	408	335	739	305	452	310	392	458	331
	Min dis	273	283	251	262	−218	271	156	5	196	273
	Avg dis	297	308	303	440	127	333	281	238	304	297
OptiTrack	Max dis	303	303	302	309	301	304	303	302	307	309
	Min dis	297	295	290	303	296	297	296	296	299	302
	Avg dis	300	300	295	305	299	302	300	298	303	305

**Figure 8:**
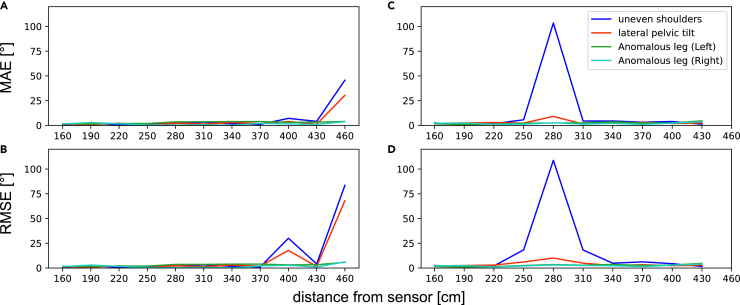
The MAE and RMSE of anterior postural indexes measured by Azure Kinect at different distances, compared with OptiTrack system’s data (A and B) without OptiTrack system interference. (C and D) with OptiTrack system interference.

Meanwhile, Figure 8 shows the mean absolute errors (MAEs) and root-mean-square errors (RMSEs) obtained comparing the human anterior posture indexes collected by Azure Kinect and OptiTrack system. The results indicate that the pose index data acquired by Azure Kinect are highly similar with those obtained by the OptiTrack system, provided there are no disturbances, up to a maximum distance of 370 cm from the human body.

The preceding figures present a visual representation of the precision and accuracy of Azure Kinect’s skeleton tracking capabilities at various distances and under different conditions. In kinematic data of key resources table, we provide the relative specific data, which can explain in more detail that Azure Kinect have the ability to evaluate postural indexes when the distance from the human body is between 160 cm and 220 cm.

#### Reliability as a static posture evaluation tool

**Figure 9:**
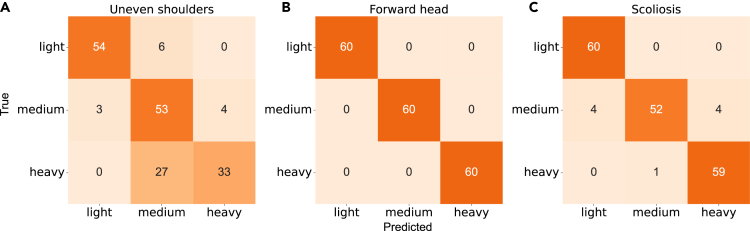
Confusion Matrix of three indexes measured by the system and the OptiTrack system (A) The confusion matrix of uneven shoulders (anterior). (B) The confusion matrix of forward head (left lateral). (C) The confusion matrix of scoliosis (posterior).

To validate the performance of our system in static postural evaluation, we compared the indexes data measured by our system with those captured by the OptiTrack optical motion capture system. As shown in Table 4 and Figure 9, we calculated Cohen’s d and ICC coefficients to analyze the mean difference and consistency between the indexes data measured by the two systems. Then, to provide a visual representation of the performance of our system, we used a confusion matrix to evaluate the indexes. In addition, we reported the accuracy and kappa coefficient of our system.

**Table 4:** Overall comparisons of posture indexes evaluated by the two systems

Indexes	Cohen’s d	ICC_2,2_ (95% CI)	Accuracy	Kappa
Uneven shoulders	0.026	0.82 (0.76–0.86)	0.78	0.74
Lateral pelvic tilt	−0.365	0.89 (0.32–0.96)	0.86	0.83
Bowlegs and knock-knees	0.417	0.88 (0.14–0.96)	0.94	0.94
Forward head	−0.007	0.97 (0.96–0.98)	1.00	1.00
Pelvic tilt	−0.158	0.98 (0.52–0.99)	1.00	1.00
Scoliosis	−0.033	0.94 (0.93–0.96)	0.95	0.94
Shoulder blade inclination	−0.061	0.85 (0.81–0.89)	0.85	0.83
p < 0.001 (for all indexes).

By analyzing Cohen’s d of the seven indexes, it showed that the mean difference is small between the indexes data measured by our system and those measured by the OptiTrack system. However, two indexes, namely lateral pelvic tilt, and bowlegs and knock-knees, exhibited a large difference. This discrepancy may be related to the precision of the hip and ankle joints themselves (shown in Figure 6). This observation was also supported by the ICC (95% CI), which showed high consistency between the indexes data measured by the two systems, except for the two indexes mentioned earlier.

Figure 9 shows the confusion matrix for three indexes - uneven shoulders (anterior), forward head (left lateral), and scoliosis (posterior) - measured by our system, compared to the OptiTrack system. Overall, our system performs well in evaluating posture indexes, but there is an issue with performance near the threshold, as shown in Figure 9A. The threshold to determine whether uneven shoulders are medium or heavy is set at 4°, and the mean angle measured by the OptiTrack system is 4.13°. However, there are some errors in the data captured by Azure Kinect, as noted in the skeleton tracking precision and accuracy section, which could cause poor performance in index evaluation near the threshold.

Finally, we have reported the accuracy and kappa coefficient of our system’s posture evaluation performance in the last two columns of Table 4, which demonstrate that the performance of our system was close to the OptiTrack system. Our system can serve as a reliable tool for initial screening of body posture abnormalities in the general population.

## Discussion

The depth sensor is a cost-effective 3D technology with numerous applications in various fields, such as industry, clinical rehabilitation, sports training, entertainment and postural control. Although there are many depth cameras available for motion capture on the market, such as Kinect series, Orbbec Astra, Femto, Leap Motion, RealSense series, ZED Stereo Camera, etc., Azure Kinect camera has a number of advantages over other cameras that make it the sensor of choice for many studies. Its features include: (1) its accuracy in capturing depth information is excellent; (2) it can be used for full-body skeleton tracking of up to six people, which expands its use scenarios, such as parent-child sports exercises at home; (3) support for many programming languages (c, C ++, python, etc.). Seven microphones are integrated, which can be combined with speech recognition. It has perfect driver and simple and easy to understand operation document, which is convenient for developers to get started quickly. The SDK is free and available. The overall barrier to development is lowered and that is why we chose it.

In this study, we have developed an Azure Kinect-based static posture assessment system for the initial screening of postural deviation in the general population. The system comprises an Azure Kinect depth camera and posture evaluation software, which was developed using the Qt framework, and boasts a user-friendly interface. The operator only needs to take three photos of the subject’s front, left side, and back while using a minimal number of markers to complete the posture assessment. Any detected issues can then be corrected by the operator.

To verify the system’s capability as a static pose evaluation tool, we first evaluated the precision and accuracy of the Azure Kinect camera in skeleton tracking at various distances, compared to an optical motion capture system (OptiTrack). We also compared the performance of our system against that of OptiTrack system during the assessment of postural indexes.

In the first experiment, we observed that the camera’s effective working range for human skeleton tracking in narrow field-of-view (NFOV) binned mode is slightly smaller than the official reference interval (50–546 cm), but is similar to the range obtained by the study of Tölgyessy et al.^41^ We noticed that the precision and accuracy of some joints’ skeleton data began to show significant fluctuations when the camera was placed 400 cm away from the human body. Such fluctuations were apparent in the Euclidean distance, depth value and X axis coordinate of the joints, as well as the depth difference measured by the camera between adjacent positions (Figures 4 and 7). Thus, we recommend that the effective working range of Azure Kinect for accurate human skeleton identification should be within 400 cm, and 370 cm is a more reliable range. However, this conclusion is only applicable to static actions, and the valid range for dynamic actions needs further verification. Additionally, it is important to make appropriate adjustments when working with subjects of varying heights. Based on the experimental results, the optimal camera distance is typically positioned to capture the subject’s entire body within the frame. With this as a guideline, an additional 10 to 30 cm can be considered acceptable.

In addition, our experiment has demonstrated the significant impact of optical infrared motion capture system on the precision and accuracy of Azure Kinect camera. However, many studies on action quality assessment (AQA) based on Azure Kinect have not reported the influence of optical systems. For instance, Albert et al. evaluated the performance of Azure Kinect in analyzing gait parameters of healthy adults at different speeds by simultaneously collecting data from both Kinect and Vicon systems.^38^ The analysis of the results showed that Azure Kinect is suitable for studying lower limb gait. Nevertheless, it is reasonable to expect that Azure Kinect would perform better without interference from optical systems, thus highlighting their potential applications in clinical rehabilitation, sports biomechanics, and other fields.

In the second experiment, we observed a strong consistency between the posture index data measured using our system and the OptiTrack system. The excellent performance on the index assessment task, as demonstrated by the confusion matrix, accuracy, and kappa coefficient, highlights the capability of our system in the initial screening of postural abnormalities. It should be noted that our system and OptiTrack show almost identical results for forward head (Cohen’s d = 0.007, ICC = 0.97 (0.96–0.98)). This consistency is in line with our observation that Azure Kinect is excellent at measuring spatial distance. However, due to the systematic errors of Azure Kinect, our system’s angle values are generally smaller than those measured by the OptiTrack system. This factor causes our system to perform moderately near the threshold of the indexes. Nevertheless, our evaluation report provides specific index values and images that can aid experts in making more accurate judgments to some extent.

On the whole, the system is a low-cost, portable, and easy-to-use tool for initial screening of abnormal posture. It can be widely used in a variety of scenarios, particularly for large-scale population screening. For instance, it can be used for body posture screening of primary and secondary school students. During this stage of continuous growth and change of the musculoskeletal system, correcting bad posture is easier.^8^ Thus, regular posture screening in schools is recommended. This can help to identify and treat posture deviations in students at an early stage. Although the use of our system requires the attachment of some auxiliary markers on the human body, this is a necessary compromise to ensure accurate assessment, as it is with all image-based assessment tools.^30^

In the future, we plan to add more functional actions, both static and dynamic, such as functional movement screen (FMS) and balance tests to the system, for a more comprehensive assessment of individual body posture and functional performance at the spatiotemporal level. Furthermore, we aim to integrate state-of-the-art AQA algorithms into our system,^42^ leveraging recent advancements in machine learning, especially supervised learning, to improve the system’s evaluation performance.

### Limitations of the study

We evaluated our system’s capability to assess static posture indexes. However, it is important to acknowledge that real people exhibit significant individual differences in body shapes, including variations in height, weight, and body composition. These differences may impact the accuracy of the system’s evaluation, as well as the camera-to-subject distance. Therefore, future studies should aim to comprehensively assess individual postural biases and functional performance by expanding posture assessments to individuals with diverse body morphological characteristics. Additionally, efforts should be made to enhance the system’s capability to evaluate a broader range of movements. Additionally, incorporating machine learning methods to improve the system’s AQA performance is a part of our future work.

## STAR★Methods

### Key resources table

**Table:** 

REAGENT or RESOURCE	SOURCE	IDENTIFIER
**Deposited data**		

Kinematic data	This paper	https://github.com/sselab2021/postureAssessment

**Software and algorithms**		

Offline processor	Github (Microsoft)	https://github.com/microsoft/Azure-Kinect-Samples/tree/master/body-tracking-samples/offline_processor
MKV data acquisition software	This paper	https://github.com/sselab2021/postureAssessment
Qt	The Qt Company	https://www.qt.io/
Qt Creator 4.6.2 (based on Qt 5.9.6, MSVC 2015)	The Qt Company	https://download.qt.io/archive/qt/5.9/5.9.6/
Inno setup compiler	Martijn Laan	https://jrsoftware.org/isdl.php
Python	Python software foundation	https://www.python.org/

### Resource availability

#### Lead contact

Further information and requests for resources should be directed and will be fulfilled by the Lead contact, Yanfei Shen (syf@bsu.edu.cn).

#### Materials availability

This study did not generate new unique reagents or materials.

### Expermental model and subject details

#### Study design

This research followed a standard control study design, with the OptiTrack system serving as the gold standard for comparison. In addition to changing the orientation of the figurine (front, side and back), the basic position of the figurine does not change. We manipulated the Azure Kinect-to-figurine distance by repositioning the Azure Kinect. In cases where the figurine’s posture remained unchanged, we separately gathered data using our system, collected data independently using the OptiTrack system, and simultaneously collected data using both systems. Subsequently, through data analysis, we assessed the potential interference between the two systems and conducted a comprehensive evaluation of the precision and accuracy of our system.

#### Figurine parameter

Our experiment required rigorous control conditions and the maintenance of a consistent posture, which can be challenging for human subjects to maintain for extended durations. Therefore, we chose to utilize a figurine as the subject throughout our experiment. Please refer to Figure S3 for a detailed image of the figurine. The figurine possessed a height of 183 cm, a shoulder width of 47 cm, and a chest circumference of 98 cm, closely resembling the dimensions of an average adult. It was mounted on a robust metal base that facilitated various postural adjustments. The figurine’s limbs were adjustable and could be secured in place using screws at the joints. Since we employed a figurine as our subject, ethical review was not deemed necessary.

### Method details

#### Design of postural evaluation system

##### Azure Kinect and computer configuration

The Azure Kinect depth camera was released by Microsoft in 2019, which is the successor of Kinect v1 and Kinect v2. The camera uses a developer kit (DK) with advanced AI sensors to build computer vision and speech models. It utilizes the ToF algorithm with amplitude modulated continuous wave (AMCW) to obtain depth information, and its specific structure is shown in Figure S1. The key features of Azure Kinect outlined in the hardware specifications section of the official documentation. For our study, the most notable feature of Azure Kinect is its excellent human skeleton tracking based on deep neural network (DNN), which identifies the spatial skeleton data of 32 joints for one body, and up to six users. We utilized Azure Kinect Software Development Kit (SDK) v1.4.1 and Azure Kinect Body Tracking SDK v1.1.2, with the NFOV binned mode selected as the operating mode. Because the study^41^^,^^43^ has demonstrated that this mode offers optimal recognition accuracy and stability.

In accordance with the official documentation, specific computer configurations are necessary for the successful operation of Azure Kinect. In our study, a laptop with the following specifications was used: (1) Intel Core i7-9750 CPU @ 2.60 GHZ; (2) 16 GB Memory; (3) NVIDIA GeForce GTX 1050; (4) USB-C port.

##### Software design

Our static postural evaluation software was developed entirely using the Qt framework, with the integrated development environment (IDE) being Qt Creator 4.6.2 (based on Qt 5.9.6, MSVC 2015). The software features a user-friendly graphical user interface (GUI) created using the Qt Widgets Module. User data is stored using a combination of SQLite database and local file storage. To generate the installation package, we utilized the windeployqt and Inno Setup compiler.

A schematic diagram of the system is shown in Figure 2. The camera and laptop are connected using a full-featured C to C cable for data transfer and power supply. The camera’s horizontal position can be calibrated using the gyroscope embedded in the camera prior to evaluation. During the evaluation, subjects are required to expose as much skin as possible (e.g., wearing tight shorts for boys, and an extra tight sports bra for girls). First, the operator pastes markers on the subject’s back for scoliosis and shoulder blade inclination, and guides the subject to stand in the middle of the camera’s field of view. Then, the operator takes three images of the subject’s front, left side, and back, with manual identification markers corrections done on the software interface when taking the back image.

The posture indexes evaluated by the system are shown in Figure 3. The skeleton data of a single frame is susceptible to outliers, therefore, we take the mean of ten frames as the input data for the index calculation. Next, we use the uneven shoulders and forward head as examples to illustrate the method of calculating the indexes by the software. As shown in Figure 3 and Table 1, the scoring criteria of uneven shoulders depends on the angle between the horizontal plane and the line of left acromion and right acromion. The angle is defined as:

(Equation 1) $$\begin{array}{c} \alpha =\arcsin\left(\frac{\left|\vec{n}\cdot \vec{V_{acr}}\right|}{\left|\vec{n}\right|\left|\vec{V_{acr}}\right|}\right) \end{array}$$

where $\vec{n}$ is the normal vector of the horizontal plane, and $\vec{V_{acr}}$ is the space vector from right to left acromion.

The scoring criteria of forward head depends on the straight-line distance between the earlobe and the acromion on the sagittal axis of the human body. The distance is defined as:

(Equation 2) $$\begin{array}{c} d=\left|X_{ear}-X_{acr}\right| \end{array}$$

where $X_{ear}$ is the X axis of the earlobe, and the $X_{acr}$ is the X axis of the acromion.

#### Validation of postural evaluation system

To assess the reliability of our system, we compared its evaluation indexes with those obtained using an optical motion capture system (OptiTrack Motion Capture System, NaturalPoint, Inc.). In the first experiment, we determined the optimal distance range between the subject and the Azure Kinect camera to minimize measurement errors. We then conducted a second experiment to validate the reliability of our system.

To simulate real-world usage, we did not deliberately warm up the Azure Kinect, even though studies suggest that the camera does not produce stable output until at least 60 min after being turned on.^43^ Moreover, because of the mutual interference between the OptiTrack motion capture system and Azure Kinect,^44^ we collected data from each system separately. Additionally, to provide reliable precision and accuracy measurements, we used a human-sized plastic figurine as subjects cannot maintain a stable posture for a prolonged period.

The experimental environment is depicted in Figure S2. Ten infrared optical motion capture cameras were positioned above the site. The figurine was placed to the left of the center of the infrared camera cluster, with its rectangular base intersecting the center line of the field vertically. The Azure Kinect was placed on a tripod 80 cm above the ground and connected to the laptop via a full-featured USB-C cable. To maintain consistent lighting conditions throughout the experiments, all lamps were kept switched on.

##### Optimal operating range of the Azure Kinect

To determine the optimal operating range of the Azure Kinect, we conducted an experiment to compare the camera’s precision and accuracy in tracking the human skeleton at different distances. We placed the figurine at a fixed position and marked 11 positions on the ground at intervals of 30 cm, starting from the closest distance (160 cm) where the Azure Kinect could capture the entire body of the figurine, up to the farthest distance of 460 cm. During the experiment, we moved only the Azure Kinect to change its distance from the figurine, while the figurine’s position remained fixed. The camera’s horizontal position was calibrated using its built-in gyroscope. To minimize measurement error, we attached a reflective marker on top of the camera to obtain the exact distance between the camera and the figurine. The experimental setup is illustrated in Figure S2.

Two different skeleton models were used for capturing human motion data in the experiments. The Plug-in Gait^45^ model, which includes 39 reflective markers, was selected as the labeling skeleton model in the OptiTrack motion capture system. The full-body marker model consisting of 32 joints was used for Azure Kinect according to the official documentation.^46^ To compute the accuracy of the Azure Kinect markers, the reflective markers from the OptiTrack system and the respective Azure Kinect skeleton markers were mapped using the method proposed by Albert et al.^38^ A subset of the OptiTrack reflective markers was mapped to the corresponding markers of the Azure Kinect. This process was achieved by assigning individual OptiTrack markers that were closest to the corresponding Azure Kinect marker, or by averaging the markers if several OptiTrack markers exist. Table S1 presents the complete marker mapping in detail.

The test process at each position comprised three stages. Firstly, the Azure Kinect was used to collect the figurine skeleton data without any reflective markers attached to the figurine. The data collection duration was set to 90 s, after which the camera was turned off. Secondly, the OptiTrack system was calibrated and the figurine was affixed with reflective markers before both systems simultaneously collected data for 90 s. Finally, the OptiTrack system collected data alone while the Azure Kinect camera was turned off. The acquisition duration was also 90 s. Each system collected data twice at every position, resulting in 22 sets of data. Our self-developed software was used to acquire the data from the Azure Kinect, which was saved as MKV video files containing image and skeleton data. The data from the OptiTrack system was acquired using Motive and exported as CSV files. These datasets were then used for further statistical analysis.

##### System reliability as postural evaluation tool

In this experiment, the Azure Kinect was positioned 190 cm from the figurine. Data for each rating grade of the indexes shown in Table 1 were collected in a specific order. The postural evaluation system and OptiTrack motion capture system were used separately for data acquisition. For example, in the uneven shoulders test, the tilt angle of the left and right acromion of the figurine was first adjusted to the angle of the light scoring criteria, and the figurine’s anterior posture index data was collected by our system a total of 60 times. Afterward, reflective markers were attached to the left and right acromion, and the OptiTrack system collected data for 10 s (OptiTrack system collection frequency is 120 Hz, and Azure Kinect’s is 30 Hz). The tilt angle of the left and right acromion was then adjusted again, and the data for the other two scoring criteria (medium and heavy) were collected. Our system collected a total of 180 sets of data for the uneven shoulders test. The same process was repeated for the other posture indexes.

### Quantification and statistical analysis

#### Skeleton tracking precision and accuracy

During data acquisition, we used the straight line of the ground as the reference for both the Azure Kinect and the OptiTrack system. Specifically, the Azure Kinect was positioned facing the figurine, while in the calibration process of the OptiTrack system, the straight line of the ground was utilized as the z axis of the geodetic coordinate system. This simplified our subsequent data processing process.

In the first experiment, the raw data was collected in MKV format by Azure Kinect. A data extraction script (offline processor) was used to extract and store the skeleton data in JSON format, resulting in 22 JSON files. From each JSON file, 1000 samples were extracted using a Python script, with equal spacing between samples. Similarly, 1000 samples were extracted from the data collected by the OptiTrack system using the same method. These samples were used to conduct statistical analysis on the precision and accuracy of the Azure Kinect at different positions.

The precision of the Azure Kinect was evaluated by calculating the Std. of the Euclidean distance, depth values, and coordinate values on the X/Y axis of the 32 joint points, as well as the anterior pose indexes captured by the camera under different scenarios with or without interference from the OptiTrack system.

As described in the previous experiment step, we did not perform a rigid transformation of the two coordinate systems. Therefore, we only discuss relative accuracy, that is, the distance difference between two adjacent positions, which was defined as:

(Equation 3) $$\begin{array}{c} relative\;accuracy={\Delta d}_{AK}-{\Delta d}_{True} \end{array}$$

where $\Delta d_{AK}$ is the distance difference between two adjacent positions measured by Azure Kinect. $\Delta d_{True}$ is the distance difference between two adjacent positions measured by OptiTrack system. In this way, the man-made error caused by moving the camera is avoided.

We evaluate the accuracy of the Azure Kinect by calculating the difference of depth values between adjacent positions and RMSE and MAE of the anterior pose indexes with or without OptiTrack system interference. No data processing filters were applied.

#### Agreement between our system and OptiTrack

To assess the level of agreement between our system and the OptiTrack system, we calculated the Cohen’s d value of the indexes. Cohen’s d is a standardized effect size used to measure the average difference between two systems, where a smaller value of d indicates a smaller difference. Cohen’s d values ranges from 0.2 to 0.5 for small effects, 0.5 to 0.8 for medium effects, and above 0.8 for large effects. Cohen’s d was computed as:

(Equation 4) $$\begin{array}{c} Cohen^{\prime }s\;d=\frac{\bar{x_{S}}-\bar{x_{O}}}{s} \end{array}$$

where $\bar{x_{s}}$ is the mean for index computed by our system, $\bar{x_{o}}$ is the mean for index computed by OptiTrack system. The denominator S is the summary Std., which was computed as:

(Equation 5) $$\begin{array}{c} S=\frac{\sqrt{\left(n_{1}-1\right){s_{1}}^{2}+\left(n_{2}-1\right){s_{2}}^{2}}}{n_{1}+n_{2}-2} \end{array}$$

where $n_{1}$ and $n_{2}$ are the respective sample sizes of the two groups.

Then, the Intraclass Correlation Coefficients (ICC) estimates and their 95% confident intervals were calculated using SPSS version 25 (SPSS Inc, Chicago, IL) based on a mean-rating (k = 2), absolute-agreement, 2-way random-effects model.^47^ ICC was computed as:

(Equation 6) $$\begin{array}{c} ICC\left(2,2\right)=\frac{MS_{R}-MS_{E}}{MS_{R}+\frac{MS_{C}-MS_{E}}{n}} \end{array}$$

where $MS_{R}$ is the mean square for rows, $MS_{E}$ is the mean square error, $MS_{C}$ is the mean square for columns and n is the number of rows.

#### Assessment of posture indexes

After calculating the angle or distance difference of the posture indexes, our system assigns a grade to each index based on the scoring criteria listed in Table 1. To further evaluate the accuracy of our system in grading the indexes, we use the confusion matrix to visualize the evaluation performance, and report the performance using accuracy and kappa coefficient. The accuracy was computed as:

(Equation 7) $$\begin{array}{c} accuracy=\frac{TP+TN}{TP+TN+FP+FN} \end{array}$$

where TP (true positive) refers to the number of positive cases that were correctly classified as positive, TN (true negative) refers to the number of negative cases that were correctly classified as negative, F P (false positive) refers to the number of negative cases that were incorrectly classified as positive, and FN (false negative) refers to the number of positive cases that were incorrectly classified as negative.

Kappa coefficient was computed as:

(Equation 8) $$\begin{array}{c} k=\frac{p_{o}-p_{e}}{1-p_{e}} \end{array}$$

where $p_{o}$ is the observed agreement proportion between the two systems, and $p_{e}$ is the expected agreement proportion of the two systems based on probability.

## Data Availability

• Kinematic data derived from human samples have been deposited at Github and Supplemental infoamation, and are publicly accessible as of the date of publication. Open access link is listed in the key resources table. • This paper does not report original code. • Any additional information required to reanalyze the data reported in this paper is available from the lead contact upon request.

## Supplemental information

Document S1. Figures S1–S3 and Table S1

Data S1. Detailed data for visualization in Figures 4 and 7, related to Figures 4 and 7
