# Supplementary material for: Design and validation of depth camera-based static posture assessment system
Source: iScience. 2023 Sep 21;26(10):107974. doi: 10.1016/j.isci.2023.107974 (PMC10551660; doi:10.1016/j.isci.2023.107974)
Supplement: Document S1. Figures S1–S3 and Table S1 [file mmc1.pdf]

iScience, Volume *26*

Supplemental information

Design and validation of depth camera-based static posture assessment system

Qingjun Xing, Ruiwei Hong, Yuanyuan Shen, and Yanfei Shen

Figure S1. Schematic of the Azure Kinect[S1] (Related to STAR Methods)

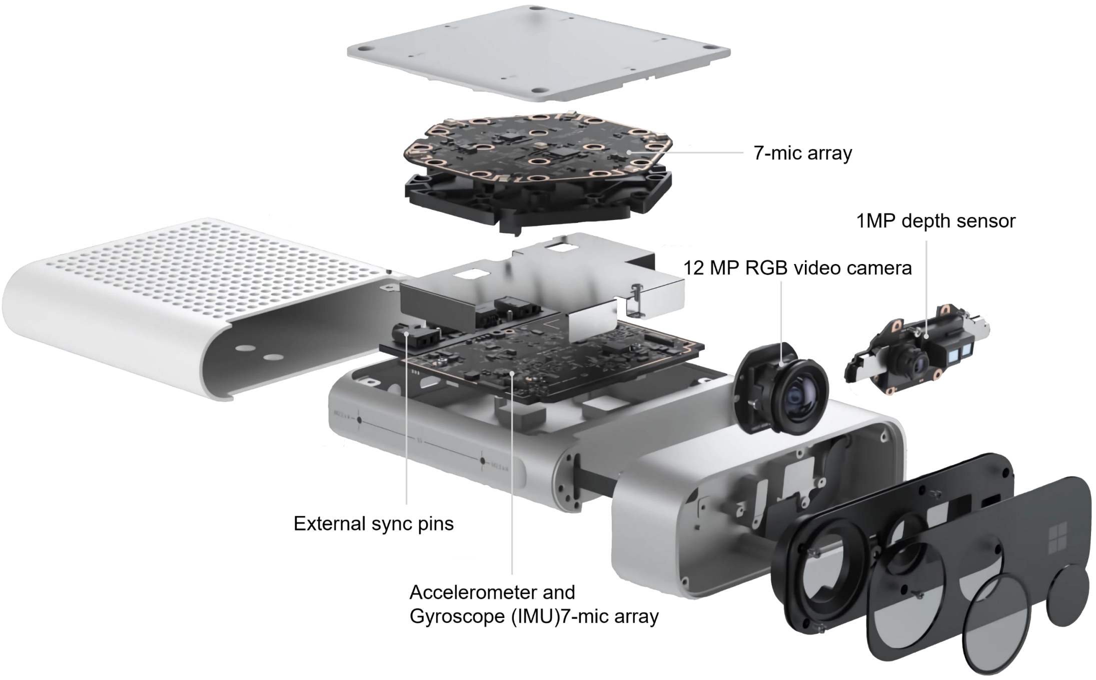

Figure S2. The experimental field (Related to STAR Methods)

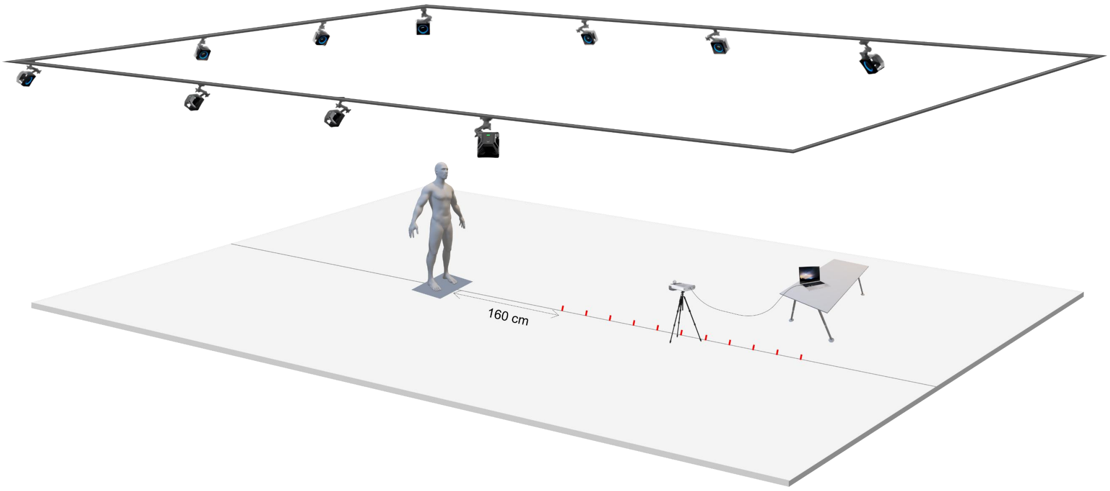

Figure S3. The image of the figurine (Related to STAR Methods)

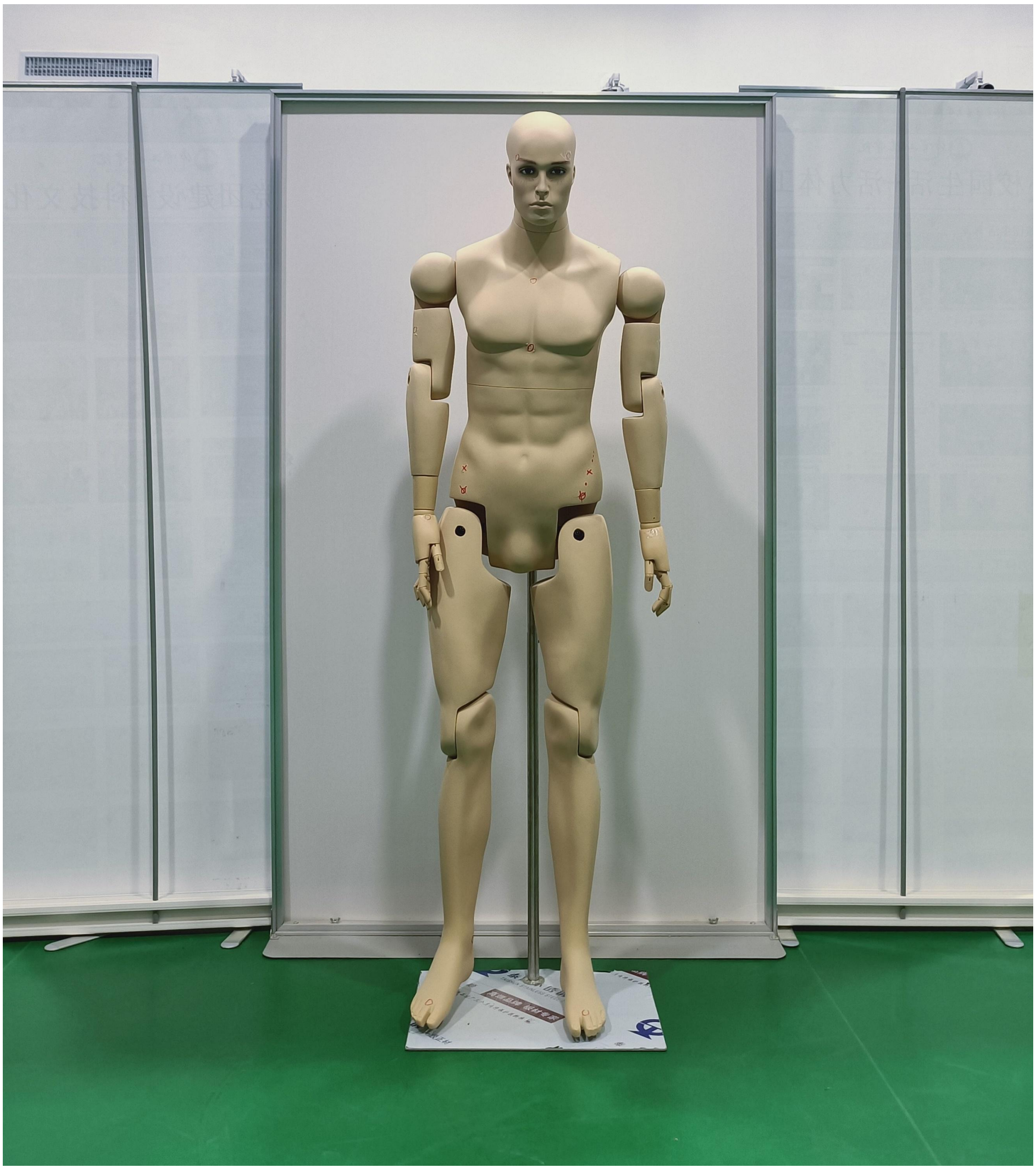

Table S1. Skeleton mapping of the 32 joints Azure Kinect model to the 39 markers OptiTrack Full body modeling with Plug-in Gait (Related to Optimal operating range of the Azure Kinect)

Index	Kinect v2/Azure Kinect Joints	Vicon Markers to Azure Kinect
01	Foot Left	LTOE
02	Foot Right	RTOE
03	Ankle Left	LANK
04	Ankle Right	RANK
05	Knee Left	LKNE
06	Knee Right	RKNE
07	Hip Left	LASI
08	Hip Right	RASI
09	Pelvis	RASI, LASI, LPSI, RPSI
10	Spine Chest	STRN
11	Spine Navel	T10
12	Head	RFHD, LFHD, RBHD, LBHD
13	Shoulder Left	LSHO
14	Shoulder Right	RSHO
15	Elbow Left	LEBL
16	Elbow Right	REBL
17	Wrist Left	LWRA + LWRB
18	Wrist Right	RWRA + RWRB
19	Hand Left	LFIN
20	Hand Right	RFIN
21	Neck	C7
22	Hand Tip Left	-
23	Hand Tip Right	-
24	Left Thumb	-
25	Right Thumb	-
26	Clavicle Right	CLAV + LSHO
27	Clavicle Left	CLAV + RSHO
28	Eye Left	-
29	Eye Right	-
30	Ear Left	-
31	Ear Right	-
32	Nose	-

References

[S1] Microsoft. Azure Kinect DK—What's inside the Azure Kinect DK. https://azure.microsoft.com/en-us/products/kinect-dk.
